# Impact of intensive hypertension criteria on multimorbidity prevalence and patterns in a multi-ethnic Chinese population

**DOI:** 10.3389/fpubh.2024.1443104

**Published:** 2024-11-29

**Authors:** Yezhou Liu, Baibing Mi, Leilei Pei, Shaonong Dang, Hong Yan, Chao Li

**Affiliations:** Department of Epidemiology and Health Statistics, School of Public Health, Xi’an Jiaotong University Health Science Center, Xi’an, Shaanxi, China

**Keywords:** multimorbidity, hypertension criteria revision, multimorbidity pattern, cardiometabolic cluster, blood pressure

## Abstract

**Background:**

The impact of intensive hypertension criteria on multimorbidity prevalence and patterns remains understudied. We investigated the prevalence and patterns of multimorbidity using both the current (140/90 mmHg) and intensive (130/80 mmHg) hypertension criteria within a multi-ethnic Chinese population.

**Methods:**

Data were obtained from the baseline survey of the Regional Ethnic Cohort Study in Northwest China, conducted from June 2018 to May 2019, which enrolled adults aged 35–74 years from five provinces. A total of 114,299 participants were included in this study. Multimorbidity was defined as the presence of at least two chronic diseases or conditions from a list of 26, ascertained through self-report and physical examination. Agglomerative hierarchical cluster analysis was employed to identify multimorbidity patterns. A hypertension-related multimorbidity pattern was identified and further analyzed. The prevalence of multimorbidity and hypertension-related pattern were analyzed in different subgroups, and subgroup cluster analyses were conducted stratified by sex, age, and ethnicity.

**Results:**

Applying the intensive 130/80 mmHg hypertension criteria resulted in an increase in multimorbidity prevalence from 17.6% (20,128 participants) to 21.7% (24,805 participants) compared to the 140/90 mmHg criteria. Four distinct multimorbidity patterns were consistently identified: cardiometabolic, digestive-bone-kidney, respiratory, and mental-cancer. Hypertension consistently clustered within the cardiometabolic pattern alongside diabetes, acute myocardial infarction, angina, and stroke/TIA, with relatively stable proportions observed even under the 130/80 mmHg threshold.

**Conclusion:**

The revision of hypertension criteria significantly expands the population identified as having multimorbidity, without altering the identified multimorbidity patterns. Hypertension commonly co-occurs within the cardiometabolic cluster. These findings highlight the need for improved treatment and management strategies specifically targeting cardiometabolic multimorbidity.

## 1 Introduction

Hypertension, a prevalent chronic condition affecting over a billion individuals globally, poses a significant public health challenge due to its strong association with various multimorbidities and comorbidities (1). Elevated blood pressure promotes atherosclerosis, left ventricular hypertrophy, and vascular damage, affecting various organs. Common coexisting conditions with hypertension include cardiovascular diseases, cerebrovascular diseases, retinopathy, peripheral vascular diseases and kidney diseases (1–4). Additionally, hypertension often co-occur with diabetes (5) and is a key component of metabolic syndrome (6), which encompasses central obesity, high blood glucose and dyslipidemia, further increasing the risk of cardiovascular disease. Studies have also demonstrated an interaction between obstructive sleep apnea (OSA) and hypertension, leading to elevated cardiovascular risk (7). These coexisting conditions not only complicate hypertension management but also substantially increase the risk of adverse health outcomes and mortality (8).

The American College of Cardiology/American Heart Association (ACC/AHA) Task Force on Clinical Practice Guidelines updated hypertension guidelines in 2017 to incorporate a lower diagnostic threshold, reducing the criteria from 140/90 mmHg to 130/80 mmHg (9), based on findings from the Systolic Blood Pressure Intervention Trial (SPRINT) (10). This study demonstrated that an intensive systolic blood pressure control target <130 mmHg across hypertensive population at high cardiovascular risk could yield cardiovascular and all-cause mortality benefits (10, 11). Subsequent researches focusing solely on hypertension indicated that while this revision expanded the diagnosed population, with those having blood pressure between 130/80 mmHg and 140/90 mmHg now labeled as having the illness, it remained cost-effective in terms of mortality reduction (12, 13). However, the effect of the revised blood pressure threshold on hypertension-related multimorbidity remains unknown. It is unclear whether this revision will bring potential challenges in managing multimorbid hypertensive patients and the need for adjustments to treatment strategies. Our team has assessed the impact and cost-effectiveness of the revised criteria within the Chinese population (14). Despite China maintaining its current hypertension threshold (15), the implication of the revised criteria on multimorbidity within this population remain uncertain, leaving open question of whether treatment strategies for multimorbid patients should be adjusted under the lower diagnostic threshold.

In this study, we investigated the prevalence rates and patterns of multimorbidity using both the current and the revised hypertension criteria based on data from the Regional Ethnic Cohort Study in Northwest China (RECS). Our aim is to gain insights into the influence of blood pressure criteria revision on the scale and characteristics of multimorbid patients.

## 2 Methods

### 2.1 Study participants

Data were obtained from the baseline survey of the RECS study, which was conducted from June 2018 to May 2019 in 13 cities or counties across five provinces or autonomous regions (Shaanxi, Xinjiang, Ningxia, Gansu, and Qinghai) in Northwest China, involving a total of 118,572 individuals. Details of design and methods have been published previously (16). In short, residents aged 35–74 years old were invited into the study. Demographic data and health-related information were gathered through face-to-face interviews conducted by trained health staff. Height, weight, waist circumference and blood pressure were also measured during the survey. Participants who provided chronic diseases information were included in the analysis. Following the application of inclusion criteria and logic examination, a final sample of 114,299 participants were included in the study analysis.

The study adhered to the principles outlined in the Declaration of Helsinki. Prior to participation, all individuals were provided with detailed information about the study’s purpose, procedures, potential risks, and benefits. Written informed consent was obtained from all participants before their enrollment in the study. Participants were assured of their right to withdraw from the study at any time without any consequences. The study protocol, including the informed consent process, was reviewed and approved by the Human Research Ethics Committee of Xi’an Jiaotong University Health Science Center (No. XJTU 2016-411).

### 2.2 Outcomes

Hypertension was diagnosed based on both blood pressure measurements and self-reported histories of hypertension. Two criteria were employed for diagnosis: the 2018 China Hypertension League (CHL) guidelines (15), which set the threshold at 140/90 mmHg, and the 2017 ACC/AHA guidelines (9), which stipulated a threshold of 130/80 mmHg.

A total of 26 diseases were included in the study. Apart from hypertension, the remaining 25 diseases were solely determined through the survey questionnaire, wherein participants were queried about whether a physician had diagnosed them with the following conditions: diabetes, acute myocardial infarction, angina, other ischemic heart diseases, stroke or transient ischemic attack (TIA), pulmonary heart disease, rheumatic heart disease, pulmonary tuberculosis, emphysema, chronic bronchitis, chronic obstructive pulmonary disease (COPD), asthma, chronic hepatitis or cirrhosis, peptic ulcer, gallstones or cholecystitis, chronic kidney disease, osteoporosis, fracture, rheumatoid arthritis, depression, anxiety, neurasthenia, other mental health disorders, traumatic brain injury, and cancer.

Multimorbidity was defined as the presence of at least two diseases from the aforementioned list within a single individual.

### 2.3 Covariate variables

The analysis considered various socio-demographic characteristics, lifestyle factors, and obesity indicators. Socio-demographic characteristics included sex, age, survey province/region, urbanicity of residence, ethnic group, education level, occupation, marital status, and wealth index tertile. The wealth index was computed using principal component analysis based on a list of self-reported family property items, and then categorized into tertiles ranging from the most economically deprived to the most affluent by district.

Lifestyle factors included smoking, drinking, physical activity, and sleep duration. Physical activity levels was evaluated using metabolic equivalents (METs), calculated from questions regarding activity types, durations, and frequencies, and subsequently grouped into tertiles by sex and age. Sleep duration was classified into three groups: < 7 h, 7–8 h, and > 8 h.

Obesity indicators included body mass index (BMI) and waist circumference. BMI was calculated using weight and height, and classified according to the Chinese obesity criteria: underweight (< 18 kg/m^2^), normal (18–23.9 kg/m^2^), overweight (24–28 kg/m^2^), and obese (≥ 28 kg/m^2^) (17). Waist circumference were used to identify central obesity, with cutoff values set at ≥85 cm for men and ≥ 80 cm for women (18).

### 2.4 Statistical analysis

Participants’ characteristics across different groups were descriptively analyzed using means and standard deviations for continuous variables, or frequency counts and percentages for categorical variables. The prevalence of multimorbidity in different groups were reported. Maps visualizing the prevalence of multimorbidity across different provinces/regions were also created.

Multimorbidity patterns were identified through agglomerative hierarchical clustering. Yule’s Q coefficients for each pair of diseases were calculated, with 1-Yule’s Q used as the dissimilarity distance for clustering analysis. The Ward’s method was applied, and a dendrogram was generated to visualize the clustering results. Multimorbidity patterns were identified and named based on the diseases present within each cluster.

Given the consistent presence of hypertension within the cardiometabolic multimorbidity pattern, further analysis focused on examining the proportions of the other four component diseases within the pattern, along with the characteristics of the cardiometabolic multimorbidity pattern (defined as having at least two conditions within the pattern). The prevalence of cardiometabolic multimorbidity in different groups were analyzed. Additionally, the frequency of combinations of hypertension and other diseases within the pattern was investigated. The proportions of the cardiometabolic pattern among all 26 diseases were also assessed.

All analyses were replicated using both hypertension criteria. Subgroup clustering analysis were conducted, stratified by sex, age (< 60 years old, ≥ 60 years old), and ethnic groups (Han, Uygur, Kazak, Hui, and Tibetan).

R (19) was utilized for the analysis. The DescTools package (20) was employed for the calculation of Yule’s Q coefficients. The Cluster package (21) was utilized for cluster analysis. The ggdendro (22) and dendextend (23) packages were employed for plotting the dendrogram.

## 3 Results

A total of 114,299 participants were included in this study. Approximately 60% were female and 40% were male, with an average age 52.8 ± 11.8 years. The participants’ basic characteristics are summarized in Table 1.

**Table 1:** Basic characteristics of participants and prevalence of multimorbidity in different subgroups according to two guideline criteria.

Characteristics	Total	2018 CHC		2017 ACC/AHA	
		No multimorbidity	Multimorbidity	No multimorbidity	Multimorbidity
Overall	114,299	94,171 (82.4)	20,128 (17.6)	89,494 (78.3)	24,805 (21.7)
Socio-demographic factors					
Sex					
Male	46,268 (40.5)	38,781 (83.8)	7,487 (16.2)	36,733 (79.4)	9,535 (20.6)
Female	68,031 (59.5)	55,390 (81.4)	12,641 (18.6)	52,761 (77.6)	15,270 (22.4)
Age, years					
Mean ± SD	52.8 ± 11.8	51.6 ± 11.7	58.4 ± 10.5	51.5 ± 11.7	57.9 ± 10.6
< 45	26,932 (23.6)	24,774 (92.0)	2,158 (8.0)	24,013 (89.2)	2,919 (10.8)
45 ~ 64	65,546 (57.3)	54,353 (82.9)	11,193 (17.1)	51,551 (78.6)	13,995 (21.4)
≥65	21,821 (19.1)	15,044 (68.9)	6,777 (31.1)	13,930 (63.8)	7,891 (36.2)
Province					
Shaanxi	44,664 (39.1)	38,926 (87.2)	5,738 (12.8)	37,083 (83.0)	7,581 (17.0)
Xinjiang	30,626 (26.8)	21,151 (69.1)	9,475 (30.9)	19,856 (64.8)	10,770 (35.2)
Ningxia	15,619 (13.7)	13,128 (84.1)	2,491 (15.9)	12,500 (80.0)	3,119 (20.0)
Gansu	20,580 (18.0)	18,485 (89.8)	2,095 (10.2)	17,710 (86.1)	2,870 (13.9)
Qinghai	2,810 (2.5)	2,481 (88.3)	329 (11.7)	2,345 (83.5)	465 (16.5)
Urbanicity					
Rural	82,535 (72.2)	67,680 (82.0)	14,855 (18.0)	64,286 (77.9)	18,249 (22.1)
Urban	31,764 (27.8)	26,491 (83.4)	5,273 (16.6)	25,208 (79.4)	6,556 (20.6)
Ethnic group					
Han	85,237 (74.8)	73,700 (86.5)	11,537 (13.5)	70,284 (82.5)	14,953 (17.5)
Uygur	15,312 (13.4)	9,181 (60.0)	6,131 (40.0)	8,582 (56.0)	6,730 (44.0)
Kazak	1,997 (1.8)	1,637 (82.0)	360 (18.0)	1,545 (77.4)	452 (22.6)
Hui	9,416 (8.3)	7,579 (80.5)	1,837 (19.5)	7,069 (75.1)	2,347 (24.9)
Tibetan	1,519 (1.3)	1,394 (91.8)	125 (8.2)	1,360 (89.5)	159 (10.5)
Other	457 (0.4)	378 (82.7)	79 (17.3)	364 (79.6)	93 (20.4)
Education level					
No formal school	21,268 (18.7)	16,318 (76.7)	4,950 (23.3)	15,279 (71.8)	5,989 (28.2)
Primary school	33,962 (29.8)	26,385 (77.7)	7,577 (22.3)	24,954 (73.5)	9,008 (26.5)
Middle school	41,184 (36.1)	35,640 (86.5)	5,544 (13.5)	34,025 (82.6)	7,159 (17.4)
College and above	17,550 (15.4)	15,541 (88.6)	2,009 (11.4)	14,962 (85.3)	2,588 (14.7)
Occupation status					
Unemployed	30,210 (26.7)	24,547 (81.3)	5,663 (18.7)	23,183 (76.7)	7,027 (23.3)
Agriculture and related workers	54,679 (48.3)	44,372 (81.1)	10,307 (18.9)	42,171 (77.1)	12,508 (22.9)
Employed	28,228 (25.0)	24,268 (86.0)	3,960 (14.0)	23,198 (82.2)	5,030 (17.8)
Marital status					
Married	100,843 (88.7)	83,970 (83.3)	16,873 (16.7)	79,818 (79.2)	21,025 (20.8)
Widowed	7,138 (6.3)	4,731 (66.3)	2,407 (33.7)	4,431 (62.1)	2,707 (37.9)
Separated/divorced	2,344 (2.1)	1,802 (76.9)	542 (23.1)	1,675 (71.5)	669 (28.5)
Never married	3,373 (3.0)	3,184 (94.4)	189 (5.6)	3,109 (92.2)	264 (7.8)
Wealth index tertiles					
1st (Deprived)	35,984 (31.9)	29,034 (80.7)	6,950 (19.3)	27,421 (76.2)	8,563 (23.8)
2nd	38,514 (34.2)	31,334 (81.4)	7,180 (18.6)	29,809 (77.4)	8,705 (22.6)
3rd (Affluent)	38,256 (33.9)	32,463 (84.9)	5,793 (15.1)	30,965 (80.9)	7,291 (19.1)
Lifestyle factors					
Smoker					
Non-smoker	92,458 (81.4)	75,508 (81.7)	16,950 (18.3)	71,796 (77.7)	20,662 (22.3)
Former smoker	3,145 (2.8)	2,299 (73.1)	846 (26.9)	2,110 (67.1)	1,035 (32.9)
Current smoker	17,919 (15.8)	15,735 (87.8)	2,184 (12.2)	14,999 (83.7)	2,920 (16.3)
Alcohol drinker					
Never or almost never	83,583 (73.5)	67,136 (80.3)	16,447 (19.7)	63,649 (76.2)	19,934 (23.8)
Occasionally	21,415 (18.8)	18,985 (88.7)	2,430 (11.3)	18,196 (85.0)	3,219 (15.0)
Usually	8,782 (7.7)	7,644 (87.0)	1,138 (13.0)	7,269 (82.8)	1,513 (17.2)
Physical activity level					
Low	38,680 (34.8)	31,033 (80.2)	7,647 (19.8)	29,454 (76.1)	9,226 (23.9)
Moderate	35,794 (32.2)	29,355 (82.0)	6,439 (18.0)	27,866 (77.9)	7,928 (22.1)
High	36,724 (33.0)	31,245 (85.1)	5,479 (14.9)	29,749 (81.0)	6,975 (19.0)
Sleep duration, hours					
< 7 h	28,973 (25.3)	22,252 (76.8)	6,721 (23.2)	20,872 (72.0)	8,101 (28.0)
7 ~ 8 h	78,301 (68.5)	65,948 (84.2)	12,353 (15.8)	62,912 (80.3)	15,389 (19.7)
> 8 h	7,025 (6.1)	5,971 (85.0)	1,054 (15.0)	5,710 (81.3)	1,315 (18.7)
BMI, kg/m^2^					
Mean ± SD	24.5 ± 3.6	24.3 ± 3.5	25.5 ± 3.9	24.3 ± 3.5	25.4 ± 3.9
Underweight	3,539 (3.1)	3,019 (85.3)	520 (14.7)	2,900 (81.9)	639 (18.1)
Normal	49,294 (43.6)	42,569 (86.4)	6,725 (13.6)	40,761 (82.7)	8,533 (17.3)
Overweight	42,164 (37.3)	34,365 (81.5)	7,799 (18.5)	32,459 (77.0)	9,705 (23.0)
Obesity	18,096 (16.0)	13,313 (73.6)	4,783 (26.4)	12,520 (69.2)	5,576 (30.8)
Waist circumference, cm					
Mean ± SD	85 ± 11	84.3 ± 10.2	89.7 ± 11.2	84.2 ± 10.2	89.1 ± 11.1
Central obesity					
Normal	38,517 (36.8)	34,341 (89.2)	4,176 (10.8)	32,995 (85.7)	5,522 (14.3)
Central obesity	66,175 (63.2)	51,913 (78.4)	14,262 (21.6)	48,936 (73.9)	17,239 (26.1)

Data were shown as *n* (%). Column % for total. Row % for two criteria.

CHL, Chinese Hypertension League; ACC, American College of Cardiology; AHA, American Heart Association; SD, Standard deviations; BMI, Body mass index.

Using the 2017 ACC/AHA criteria, the prevalence of multimorbidity increased from 17.6% (20,128) to 21.7% (24,805). The prevalence of multimorbidity was higher among certain groups, including females, older individuals, residents of Xinjiang and rural areas, those of Uygur ethnicity, individuals with lower education levels (no formal schooling and primary schooling), those unemployed and engaging in agriculture or related work, as well as those who were widowed, separated or divorced, economically disadvantaged, non-smokers or former smokers, never or almost never drinkers, physically inactive, and individuals who slept less than 7 h. Additionally, individuals with higher BMI levels, and higher waist circumference also had a higher prevalence of multimorbidity.

When applying the 2017 ACC/AHA criteria, the characteristics of individuals with multimorbidity changed slightly. Younger individuals (under 65 years old), Han ethnicity participants, those with higher education levels, employed individuals, those who were married or never married, more affluent participants, current smokers, occasional drinkers, and those with normal BMI and waist circumference showed an increment in the prevalence of multimorbidity. Prevalence maps illustrating regional disparities are presented in Supplementary Figure 1.

### 3.1 Multimorbidity patterns

**Figure 1:**
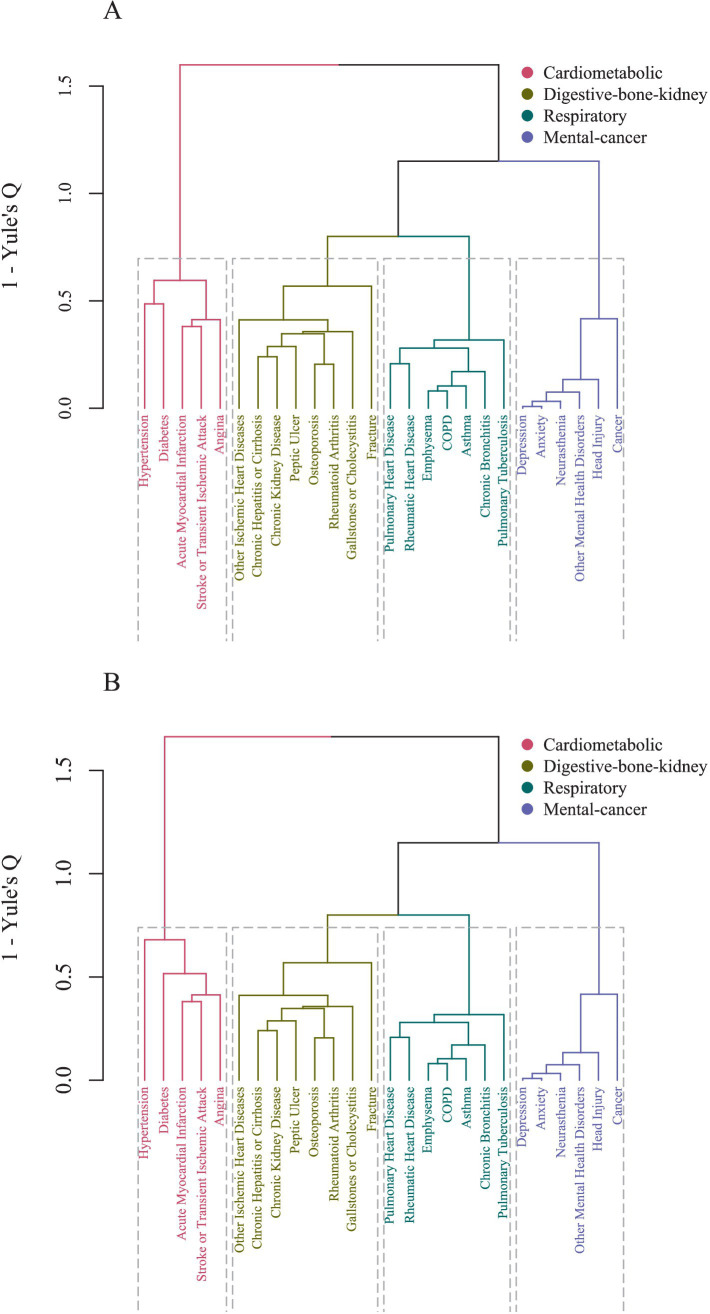
**(A)** Dendrogram of cluster analysis for 26 diseases according to 2018 CHL hypertension guideline criteria. **(B)** Dendrogram of cluster analysis for 26 diseases according to 2017 ACC/AHA hypertension guideline criteria. CHL, Chinese Hypertension League; ACC, American College of Cardiology; AHA, American Heart Association; COPD, Chronic Obstructive Pulmonary Disease.

Clustering analysis revealed four multimorbidity patterns within the population. Following the application of the 2017 ACC/AHA criteria, the overall structure remained unchanged. Dendrograms illustrated these clusters are depicted in Figure 1. The identified patterns were cardiometabolic, digestive-bone-kidney, respiratory and mental-cancer. Notably, hypertension consistently clustered with diabetes, acute myocardial infarction, angina, and stroke/TIA within the cardiometabolic multimorbidity pattern.

### 3.2 Cardiometabolic pattern

Cardiometabolic multimorbidity effected 6.4% participants as application of the 2018 CHL criteria, which increased to 8% following the application of the lowered 2017 ACC/AHA criteria (Table 2).

**Table 2:** Prevalence of cardiometabolic multimorbidity according to two guideline criteria.

Characteristic	2018 CHL		2017 ACC/AHA	
	No	Yes	No	Yes
Overall	106,954 (93.6)	7,345 (6.4)	105,150 (92.0)	9,149 (8.0)
Socio-demographic factors				
Sex				
Male	43,271 (93.5)	2,997 (6.5)	42,433 (91.7)	3,835 (8.3)
Female	63,683 (93.6)	4,348 (6.4)	62,717 (92.2)	5,314 (7.8)
Age, years				
Mean ± SD	52.2 ± 11.7	61.8 ± 8.7	52.1 ± 11.7	61.4 ± 8.9
< 45	26,667 (99.0)	265 (1.0)	26,547 (98.6)	385 (1.4)
45 ~ 64	61,743 (94.2)	3,803 (5.8)	60,673 (92.6)	4,873 (7.4)
≥ 65	18,544 (85.0)	3,277 (15.0)	17,930 (82.2)	3,891 (17.8)
Province				
Shaanxi	42,462 (95.1)	2,202 (4.9)	41,758 (93.5)	2,906 (6.5)
Xinjiang	27,359 (89.3)	3,267 (10.7)	26,786 (87.5)	3,840 (12.5)
Ningxia	14,495 (92.8)	1,124 (7.2)	14,240 (91.2)	1,379 (8.8)
Gansu	19,946 (96.9)	634 (3.1)	19,714 (95.8)	866 (4.2)
Qinghai	2,692 (95.8)	118 (4.2)	2,652 (94.4)	158 (5.6)
Urbanicity				
Rural	77,444 (93.8)	5,091 (6.2)	76,174 (92.3)	6,361 (7.7)
Urban	29,510 (92.9)	2,254 (7.1)	28,976 (91.2)	2,788 (8.8)
Ethnic group				
Han	80,359 (94.3)	4,878 (5.7)	79,008 (92.7)	6,229 (7.3)
Uygur	13,795 (90.1)	1,517 (9.9)	13,592 (88.8)	1,720 (11.2)
Kazak	1,942 (97.2)	55 (2.8)	1,923 (96.3)	74 (3.7)
Hui	8,594 (91.3)	822 (8.7)	8,382 (89.0)	1,034 (11.0)
Tibetan	1,499 (98.7)	20 (1.3)	1,492 (98.2)	27 (1.8)
Other	427 (93.4)	30 (6.6)	421 (92.1)	36 (7.9)
Education level				
No formal school	19,297 (90.7)	1,971 (9.3)	18,860 (88.7)	2,408 (11.3)
Primary school	31,474 (92.7)	2,488 (7.3)	30,937 (91.1)	3,025 (8.9)
Middle school	38,829 (94.3)	2,355 (5.7)	38,138 (92.6)	3,046 (7.4)
College and above	17,030 (97.0)	520 (3.0)	16,893 (96.3)	657 (3.7)
Occupation status				
Unemployed	27,647 (91.5)	2,563 (8.5)	27,031 (89.5)	3,179 (10.5)
Agriculture & related workers	51,281 (93.8)	3,398 (6.2)	50,480 (92.3)	4,199 (7.7)
Employed	26,899 (95.3)	1,329 (4.7)	26,520 (93.9)	1,708 (6.1)
Marital status				
Married	94,647 (93.9)	6,196 (6.1)	93,042 (92.3)	7,801 (7.7)
Widowed	6,184 (86.6)	954 (13.4)	6,045 (84.7)	1,093 (15.3)
Separated/divorced	2,203 (94.0)	141 (6.0)	2,162 (92.2)	182 (7.8)
Never married	3,349 (99.3)	24 (0.7)	3,339 (99.0)	34 (1.0)
Wealth index tertiles				
1st (Deprived)	33,434 (92.9)	2,550 (7.1)	32,786 (91.1)	3,198 (8.9)
2nd	36,079 (93.7)	2,435 (6.3)	35,487 (92.1)	3,027 (7.9)
3rd (Affluent)	35,949 (94.0)	2,307 (6.0)	35,396 (92.5)	2,860 (7.5)
Lifestyle factors				
Smoker				
Non-smoker	86,382 (93.4)	6,076 (6.6)	84,974 (91.9)	7,484 (8.1)
Former smoker	2,762 (87.8)	383 (12.2)	2,661 (84.6)	484 (15.4)
Current smoker	17,085 (95.3)	834 (4.7)	16,804 (93.8)	1,115 (6.2)
Alcohol drinker				
Never or almost never	77,449 (92.7)	6,134 (7.3)	75,996 (90.9)	7,587 (9.1)
Occasionally	20,623 (96.3)	792 (3.7)	20,380 (95.2)	1,035 (4.8)
Usually	8,402 (95.7)	380 (4.3)	8,303 (94.5)	479 (5.5)
Physical activity level tertiles				
Low	35,635 (92.1)	3,045 (7.9)	34,958 (90.4)	3,722 (9.6)
Moderate	33,393 (93.3)	2,401 (6.7)	32,807 (91.7)	2,987 (8.3)
High	35,023 (95.4)	1,701 (4.6)	34,519 (94.0)	2,205 (6.0)
Sleep duration, hours				
< 7 h	26,294 (90.8)	2,679 (9.2)	25,714 (88.8)	3,259 (11.2)
7 ~ 8 h	74,061 (94.6)	4,240 (5.4)	72,936 (93.1)	5,365 (6.9)
> 8 h	6,599 (93.9)	426 (6.1)	6,500 (92.5)	525 (7.5)
BMI, kg/m^2^				
Mean ± SD	24.4 ± 3.6	26.0 ± 3.8	24.4 ± 3.6	25.8 ± 3.7
Underweight	3,427 (96.8)	112 (3.2)	3,383 (95.6)	156 (4.4)
Normal	47,224 (95.8)	2,070 (4.2)	46,563 (94.5)	2,731 (5.5)
Overweight	39,063 (92.6)	3,101 (7.4)	38,331 (90.9)	3,833 (9.1)
Obesity	16,164 (89.3)	1,932 (10.7)	15,822 (87.4)	2,274 (12.6)
Waist circumference, cm				
Mean ± SD	84.9 ± 10.5	90.8 ± 10.9	84.8 ± 10.5	90.2 ± 10.8
Central obesity				
Normal	37,244 (96.7)	1,273 (3.3)	36,780 (95.5)	1,737 (4.5)
Central Obesity	60,601 (91.6)	5,574 (8.4)	59,366 (89.7)	6,809 (10.3)

Data were shown as *n* (row %).

CHL, Chinese Hypertension League; ACC, American College of Cardiology; AHA, American Heart Association; SD, Standard deviations; BMI, Body mass index.

**Figure 2:**
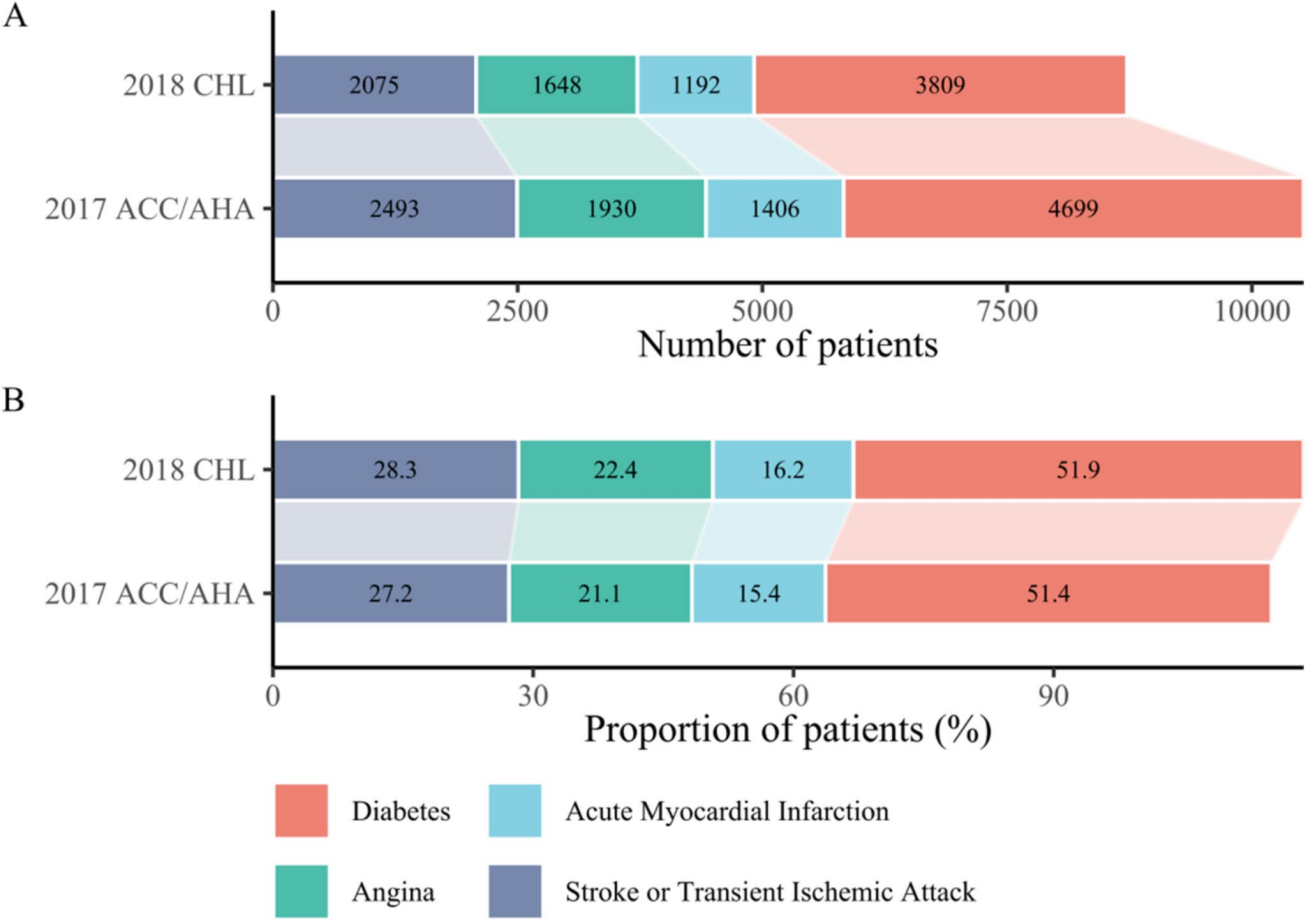
**(A)** Number of cases of the four component diseases (excluding hypertension) among participants with cardiometabolic multimorbidity under two guideline criteria. Cases increased due to adoption of the intensive criteria for hypertension. **(B)** Proportion of each of the four component diseases (excluding hypertension) among participants with cardiometabolic multimorbidity under two guideline criteria. Relative proportions decreased for other diseases due to increased hypertension cases, with no change in rank order. CHL, Chinese Hypertension League; ACC, American College of Cardiology; AHA, American Heart Association.

Further analysis of the other four diseases within the pattern revealed unexpected increases in numbers, accompanied by decreases in proportions. However, the proportions among these diseases remained consistent, with diabetes accounting for approximately half and stroke for about a third. These finding are presented in Figure 2.

In the cardiometabolic pattern, the most common disease combinations were hypertension and diabetes (3,595, 3.15%), followed by hypertension and stroke (1924, 1.68%). Conversely, the least common combination involved all five diseases, affecting only six patients (Supplementary Table 1).

### 3.3 Characteristics of cardiometabolic multimorbidity

The analysis of cardiometabolic multimorbidity prevalence across different groups is detailed in Table 2. The prevalence of cardiometabolic multimorbidity was higher among individuals who were older, resided in Xinjiang and Ningxia, were of Uygur and Hui ethnicity, had lower education levels (no formal schooling and primary schooling), were unemployed, widowed, economically disadvantaged, non-smokers and former smokers, never or almost never drinkers, were physically inactive, slept less than 7 h, and had higher BMI levels and waist circumferences.

When applying the 2017 ACC/AHC criteria, there was a slight increase of 1.6% in the population with cardometabolic multimorbidity. In this revised population, the prevalence was higher among males, younger individuals, residents of Shaanxi and Gansu, individuals of Han ethnicity, higher education levels (middle school and above), fewer widowed individuals, more current smokers and occasional or regular smokers, individuals with high physical activity levels, those who slept between 7 and 8 h, and those with normal BMI and waist circumference.

The prevalence of cardiometabolic multimorbidity varied among the 26 diseases, ranging from 8.4% in depression to 69.6% in acute myocardial infarction when applying the 2018 CHL criteria. Upon application of the 2017 ACC/AHA criteria, the prevalence increased in 25 diseases except hypertension, ranging from 9.2% in depression to 82.8% in diabetes. Notably, in hypertension, the prevalence of the cardiometabolic pattern decreased from 16.6 to 13%. These findings are depicted in Supplementary Figure 4.

### 3.4 Subgroup clustering analysis

Subgroup clustering analyses were conducted, stratified by sex, age, and ethnic groups. Dendrograms are presented in Supplementary Figures 2–4. The application of two hypertension criteria did not change multimorbidity pattern across different subgroups.

Although slightly differences were observed in male and female participants, hypertension consistently clustered within the cardiometabolic pattern. The respiratory and mental patterns remained stable.

Across different age groups, the cardiometabolic, respiratory and mental pattern were also well-replicated. However, in participants under 60 years old, the cardiometabolic pattern comprised only four diseases, while in those over 60, it included five diseases.

Disparities among different ethnicity groups were substantial. The multimorbidity patterns in Han individuals were in line with those of the overall population. In Uygurs, hypertension, diabetes and acute myocardial infarction clustered together, while the respiratory and mental patterns remained stable. In Hui individuals, the cardiometabolic and respiratory patterns were distinctive. However, in Kazak and Tibetan individuals, it was challenging to identify a specific pattern.

## 4 Discussion

In this study, we observed a notable increase in the prevalence of multimorbidity among adults aged 35–75 years old from 17.6 to 21.7% when applying the 130/80 mmHg threshold for hypertension, This resulted in an absolute increment of nearly 4,700 patients. However, the revised hypertension criteria did not significantly alter the predominant multimorbidity patterns in the study population, which remained cardiometabolic, digestive-bone-kidney, respiratory, and mental-cancer. Consistently, hypertension was clustered within the cardiometabolic pattern alongside diabetes, acute myocardial infarction, stroke/TIA, and angina, using both hypertension criteria. The revised criteria led to an expansion of the cardiometabolic multimorbidity group (from 6.4% to 8%), while the proportional contribution of each disease within the multimorbidity pattern remained stable.

Previous studies conducted in Chinese population have reported a wide range of multimorbidity prevalence rates, from 20.6 to 66.7%, which can be attributed to differences in the diseases included in the analyses and different study populations (24). Our study revealed that multimorbidity affects approximately one fifth of the population in Northwest China, emphasizing the need of incorporating multimorbidity into healthcare planning. The China Kadoorie Biobank (CKB) study has reported that the prevalence of multimorbidity has increased by 25% over an 8-year period in middle-aged people (25), highlighting the urgent need for interventions to mitigate the impending rise in multimorbidity. Consistent with prior research (26, 27), our study identified women, older individuals, living in rural areas, those with lower education levels, and those of lower socioeconomic status as vulnerable groups of multimorbidity. Race or ethnic disparity have been reported in the United States and the United Kingdom population (28, 29), few studies in Chinese did so. We observed large difference of multimorbidity distribution across regions and ethnic groups, those living in Xinjing and Uygur ethnic group were predisposed to having multimorbidity. The role of obesity in multimorbidity has been debated, with some studies considering it as a disease and others as a risk factor (25, 30, 31). In this study, we treated obesity as a risk factor and both general and central obesity were associated with an increased risk of multimorbidity.

There is currently no consensus on the optimal method for identifying multimorbidity patterns, with previous studies employing various techniques, such as clustering (25, 30, 32), association rules mining (33–35), exploratory factor analysis (36–38), network analysis (28, 35, 39) and latent class analysis (32, 40, 41). Although specific patterns varied across methods, some patterns, such as cardiometabolic and respiratory patterns, were relatively stable (32, 40, 42). Similarly, our subgroup analyses consistently replicated the cardiometabolic and respiratory patterns, with only minor discrepancies noted.

Using both hypertension criteria, our study consistently clustered hypertension within the cardiometabolic pattern. These findings are consistent with existing literature and highlight the complex relationship between hypertension and cardiovascular metabolic disorders. Hypertension and cardiovascular metabolic diseases share numerous common risk factors, including age, genetic predisposition, unhealthy lifestyle (e.g., high salt intake, physical inactivity, and smoking), and metabolic disturbances such as obesity and insulin resistance (43, 44). These factors collectively promote pathological processes like atherosclerosis, inflammation, and oxidative stress, leading to the development and progression of both hypertension and cardiovascular metabolic diseases (45). Chronic hypertension damages vascular endothelium, promoting the development and progression of atherosclerosis, increasing the risk of myocardial infarction, stroke/TIA, and angina (46, 47). Additionally, hypertension can lead to left ventricular hypertrophy and myocardial remodeling, increasing the risk of heart failure (48, 49). Metabolic disturbances such as diabetes and dyslipidemia exacerbate atherosclerosis and vascular damage while increasing the risk of thrombosis, further elevating the incidence of cardiovascular events (50–53). Hypertension activates the renin-angiotensin-aldosterone system (RAAS) (54) and the sympathetic nervous system (55), contributing to vasoconstriction, sodium and water retention, and myocardial hypertrophy, further exacerbating hypertension and cardiovascular disease progression. Given the close relationship between hypertension and cardiovascular metabolic diseases, early identification and intervention for hypertension and associated metabolic abnormalities are crucial. A comprehensive cardiovascular risk assessment and a multi-faceted management approach are essential for hypertensive patients, incorporating lifestyle interventions, pharmacological therapy, and control of metabolic parameters such as blood glucose and lipids. Public health education should be strengthened to promote healthy lifestyles and prevent the onset of hypertension and cardiovascular metabolic diseases.

The revised criteria resulted in an expansion of both the hypertensive patient group and the cardiometabolic multimorbidity group, while the proportional contribution of each disease within the multimorbidity pattern remained stable. The increase in hypertensive patients encompassed those with blood pressure between 130/80 mmHg and 140/90 mmHg, previously considered pre-hypertensive. Similarly, the expansion of the cardiometabolic multimorbid group included patients with only one of the following conditions: diabetes, acute myocardial infarction, stroke/TIA, or angina, under the 140/90 mmHg threshold. Several studies, including our own, have demonstrated the cost-effectiveness of intensive management despite the increased patient population (12–14). However, there remains a gap in health economic evaluations specifically for multimorbid hypertensive patients using the lower blood pressure threshold. While the expanded patient group may increase the workload associated with cardiometabolic multimorbidity management, the stable disease composition suggests that the core principles for revising guidelines on multimorbid hypertension remain relevant. The focus should shift toward improving blood pressure control rates and adherence to recommended treatment regimens. Multimorbid hypertensive patients may need more intensive control targets and tailored management strategies. Existing guidelines offer management strategies for hypertension with comorbidities such as coronary heart disease, diabetes, and stroke (9, 15). However, these recommendations are often limited to single comorbidities. Management protocols for patients with complex multimorbidity require further development and refinement.

This study has limitations inherent to its cross-sectional design, preventing the exploration of causal relationships among multimorbidity diseases. The non-random sampling method introduces potential selection bias, and the reliance on self-reported data for diseases other than hypertension raises concerns about recall bias and underreporting. Additionally, the selection of diseases was not comprehensive, limiting the analysis to the specific conditions captured in the survey. The class imbalance between the multimorbidity and non-multimorbidity groups may also limit the generalizability of the findings. Although subgroup analyses by sex and age showed consistent results with the main findings, significant disparities were observed in the analysis by ethnicity. These findings suggest caution when interpreting results across broader populations. Despite these limitations, the large and diverse sample size provides valuable insights into the prevalence and patterns of multimorbidity within this population.

In conclusion, our study demonstrates that revised hypertension criteria expands the multimorbidity patient population without altering established patterns. Hypertension remains consistently grouped within the cardiometabolic pattern, alongside diabetes, acute myocardial infarction, stroke/TIA, and angina, with relatively stable proportions under the 130/80 mmHg threshold. These results underscore the need for improved treatment and management strategies targeting cardiometabolic multimorbidity. Further research should investigate the long-term health outcomes and cost-effectiveness implications of managing multimorbidity in the context of the revised hypertension guidelines. Longitudinal studies are particularly needed to elucidate the dynamic interplay between hypertension and other comorbid conditions, as well as the potential impact on patient prognosis and healthcare utilization. Ultimately, a comprehensive understanding of multimorbidity patterns and their clinical and economic consequences is crucial to inform the development of more effective, patient-centered approaches to chronic disease management.

## Data Availability

The datasets presented in this article are not readily available because the data contains sensitive information. Requests to access the datasets should be directed to Chao Li, lcxjtu@xjtu.edu.cn and Hong Yan, yanhonge@mail.xjtu.edu.cn.
