# Supplementary material for: Impact of intensive hypertension criteria on multimorbidity prevalence and patterns in a multi-ethnic Chinese population
Source: Front Public Health. 2024 Nov 29;12:1443104. doi: 10.3389/fpubh.2024.1443104 (PMC11638201; doi:10.3389/fpubh.2024.1443104)
Supplement: Supplementary file 1 [file Data_Sheet_1.docx]

Supplementary Material

# Supplementary Figures and Tables

## Supplementary Figures


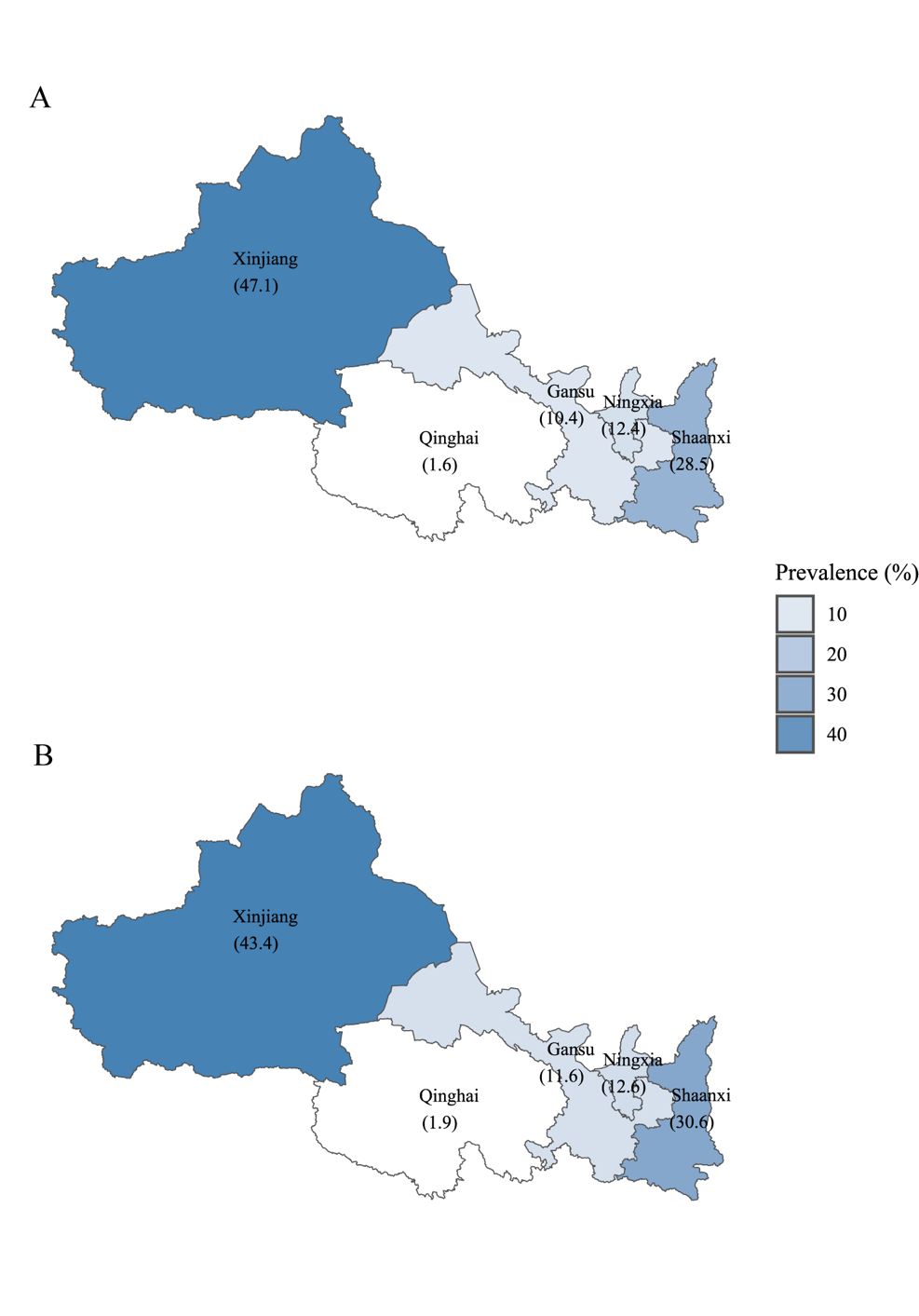


**Supplementary Figure 1 Multimorbidity prevalences of five provinces/regions in Northwest China according to two hypertension criteria.** (A) 2018 CHL, (B) 2017 ACC/AHA. CHL = Chinese Hypertension League, ACC = American college of Cardiology, AHA = American heart Association

**Supplementary Figure 2 Subgroup dendrogram of cluster analysis stratified by sex.** CHL = Chinese Hypertension League, ACC = American college of Cardiology, AHA = American heart Association, COPD = Chronic Obstructive Pulmonary Disease

**Supplementary Figure 3 Subgroup dendrogram of cluster analysis stratified by age.** CHL = Chinese Hypertension League, ACC = American college of Cardiology, AHA = American heart Association, COPD = Chronic Obstructive Pulmonary Disease.

**Supplementary Figure 4 Subgroup dendrogram of cluster analysis stratified by ethnic group.** CHL = Chinese Hypertension League, ACC = American college of Cardiology, AHA = American heart Association, COPD = Chronic Obstructive Pulmonary Disease.

**Supplementary Figure 5 Proportion of cardiometabolic multimorbidity in 26 diseases** CHL = Chinese Hypertension League, ACC = American college of Cardiology, AHA = American heart Association, COPD = Chronic Obstructive Pulmonary Disease.

## Supplementary Table

| Supplementary Table 1 Co-occurrence of hypertension and other four diseases in cardiometabolic multimorbidity patterns among all participants according to two guideline criteria | | | | | | | | | |
| --- | --- | --- | --- | --- | --- | --- | --- | --- | --- |
| **Cardiometabolic disease combination** | | | | | **Number of disease** | **2018 CHL** | | **2017 ACC/AHA** | |
| **Hypertension** | **Diabetes** | **Acute Myocardial Infarction** | **Angina** | **Stroke or Transient Ischemic Attack** |  | **Count** | **%** | **Count** | **%** |
| 1 | 1 |  |  |  | 2 | 3595 | 3.145 | 4580 | 4.007 |
| 1 |  |  |  | 1 | 2 | 1924 | 1.683 | 2406 | 2.105 |
| 1 |  |  | 1 |  | 2 | 1474 | 1.290 | 1827 | 1.598 |
| 1 |  | 1 |  |  | 2 | 1038 | 0.908 | 1329 | 1.163 |
| 1 | 1 |  |  | 1 | 3 | 290 | 0.254 | 317 | 0.277 |
| 1 | 1 |  | 1 |  | 3 | 266 | 0.233 | 304 | 0.266 |
| 1 | 1 | 1 |  |  | 3 | 190 | 0.166 | 232 | 0.203 |
| 1 |  |  | 1 | 1 | 3 | 162 | 0.142 | 182 | 0.159 |
| 1 |  | 1 |  | 1 | 3 | 130 | 0.114 | 153 | 0.134 |
| 1 |  | 1 | 1 |  | 3 | 88 | 0.077 | 111 | 0.097 |
| 1 | 1 |  | 1 | 1 | 4 | 38 | 0.033 | 40 | 0.035 |
| 1 | 1 | 1 | 1 |  | 4 | 35 | 0.031 | 42 | 0.037 |
| 1 | 1 | 1 |  | 1 | 4 | 27 | 0.024 | 30 | 0.026 |
| 1 |  | 1 | 1 | 1 | 4 | 13 | 0.011 | 14 | 0.012 |
| 1 | 1 | 1 | 1 | 1 | 5 | 6 | 0.005 | 6 | 0.005 |
| CHL = Chinese Hypertension League, ACC = American college of Cardiology, AHA = American heart Association, COPD = Chronic Obstructive Pulmonary Disease. | | | | | | | | | |
